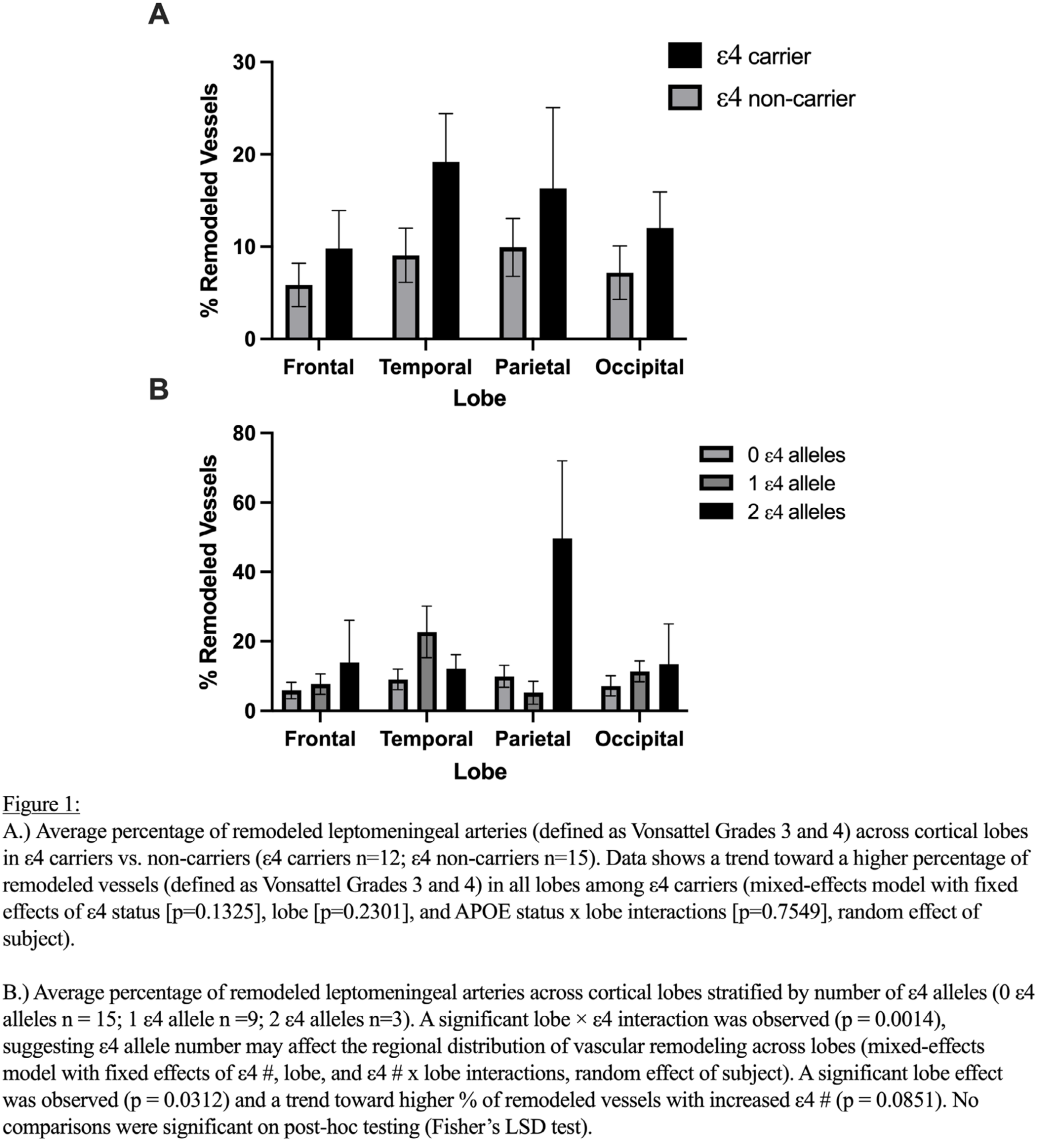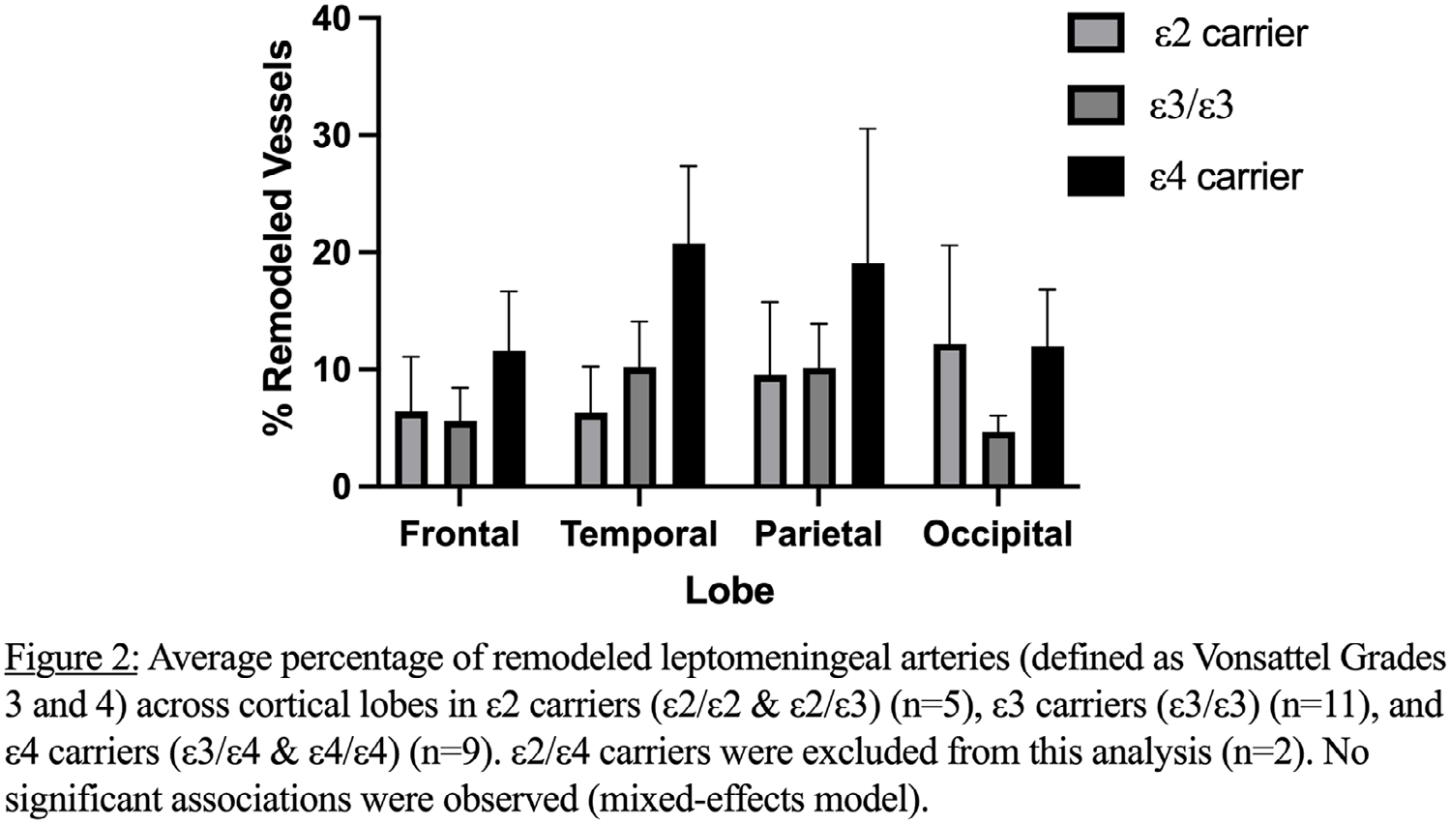# The Role of APOE in Leptomeningeal Vascular Remodeling in CAA

**DOI:** 10.1002/alz70861_108229

**Published:** 2025-12-23

**Authors:** S. Soumya Vytla, Orla Bonnar, Kali Vom Eigen, Corinne A. Auger, Susanne J. van Veluw, Mariel Kozberg

**Affiliations:** ^1^ Massachusetts General Hospital, Harvard Medical School, Boston, MA USA; ^2^ Massachusetts General Hospital, Cambridge, MA USA; ^3^ MassGeneral Institute for Neurodegenerative Disease, Massachusetts General Hospital, Harvard Medical School, Charlestown, MA USA; ^4^ Massachusetts General Hospital, Boston, MA USA

## Abstract

**Background:**

Cerebral amyloid angiopathy (CAA) is a small vessel disease marked by amyloid‐β (Aβ) accumulation in cortical and leptomeningeal vessels, often co‐occurring with Alzheimer’s disease (AD), and is a leading cause of intracerebral hemorrhage. APOE ε4 (ε4) increases risk for CAA, AD, and amyloid‐related imaging abnormalities (ARIA)—Aβ–immunotherapy–associated complications. Notably, CAA diverges from AD in that APOE ε2 (ε2), protective in AD, has been associated with increased risk of CAA‐related hemorrhage. However, APOE’s role in CAA progression is unclear. Given evidence linking leptomeningeal remodeling to larger CAA‐related hemorrhages, we examined its relationship with APOE allele status in autopsied brains of clinically diagnosed CAA patients.

**Method:**

Samples from frontal, parietal, temporal, and occipital lobes were collected from 28 CAA patients. APOE status was determined in 27/28 via DNA extraction from FFPE sections and genotyping at the MGB Biobank; ε4 IHC confirmed positivity. Sections were stained for Aβ and H&E, digitized, and an 8 mm² leptomeningeal region was selected for artery grading using the Vonsattel CAA scale, blinded to genotype. A second blinded rater assessed a subset (interrater reliability: 93.7%).

**Result:**

ε2 (22.8%) and ε4 (44.4%) alleles were over‐represented in our cohort compared to population norms. ε4 carriers showed a trend toward increased leptomeningeal artery remodeling (mixed‐effects model, *p* =0.1325, Figure 1A). Remodeling rates trended toward increasing with ε4 allele number (*p* =0.0851), and a significant lobe × ε4 interaction was observed (*p* =0.0014, mixed‐effects model, Figure 1B). ε2 carriers did not show greater remodeling overall, though there was a numerically higher percentage of remodeling in the occipital lobe vs. ε3/ε3 carriers (Figure 2). No remodeling was seen in the single ε2/ε2 case.

**Conclusion:**

Our findings suggest vascular remodeling is more prominent in ε4 carriers, even within a cohort of CAA patients, implicating a role in ε4‐related CAA pathology and ARIA risk. We will present ongoing work examining vascular remodeling features across APOE genotypes. ε2 was notably overrepresented in our cohort. Although we found no significant link between ε2 and remodeling, ε2/ε3 status did not appear protective, with remodeling comparable to—or exceeding—that in ε3/ε3 cases.